# Supplementary material for: Single-nucleus transcriptomics reveal the cytological mechanism of conjugated linoleic acids in regulating intramuscular fat deposition
Source: eLife. 2025 Mar 7;13:RP99790. doi: 10.7554/eLife.99790 (PMC11888599; doi:10.7554/eLife.99790)
Supplement: Figure 7—source data 1. — The lower membranes correspond to CON group and higher membranes correspond to CLA group. [file elife-99790-fig7-data1.pdf]

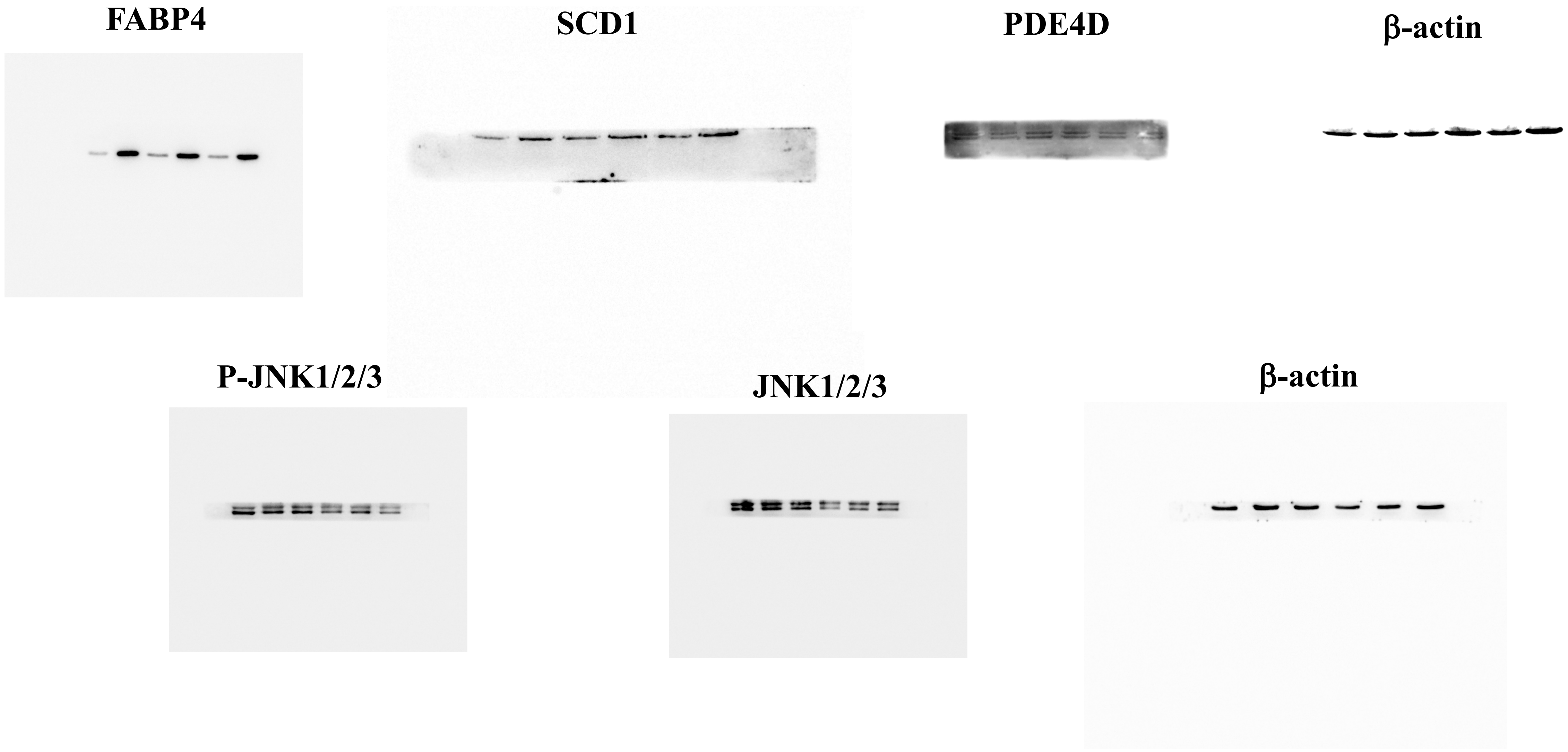

Figure 7-Source Data 1. Original membranes corresponding to Figure 7D and 7G. The lower membranes correspond to CON group and higher membranes correspond to CLA group.
