# Supplementary figures and images for: Single-nucleus transcriptomics reveal the cytological mechanism of conjugated linoleic acids in regulating intramuscular fat deposition
Source: eLife. 2025 Mar 7;13:RP99790. doi: 10.7554/eLife.99790 (PMC11888599; doi:10.7554/eLife.99790)

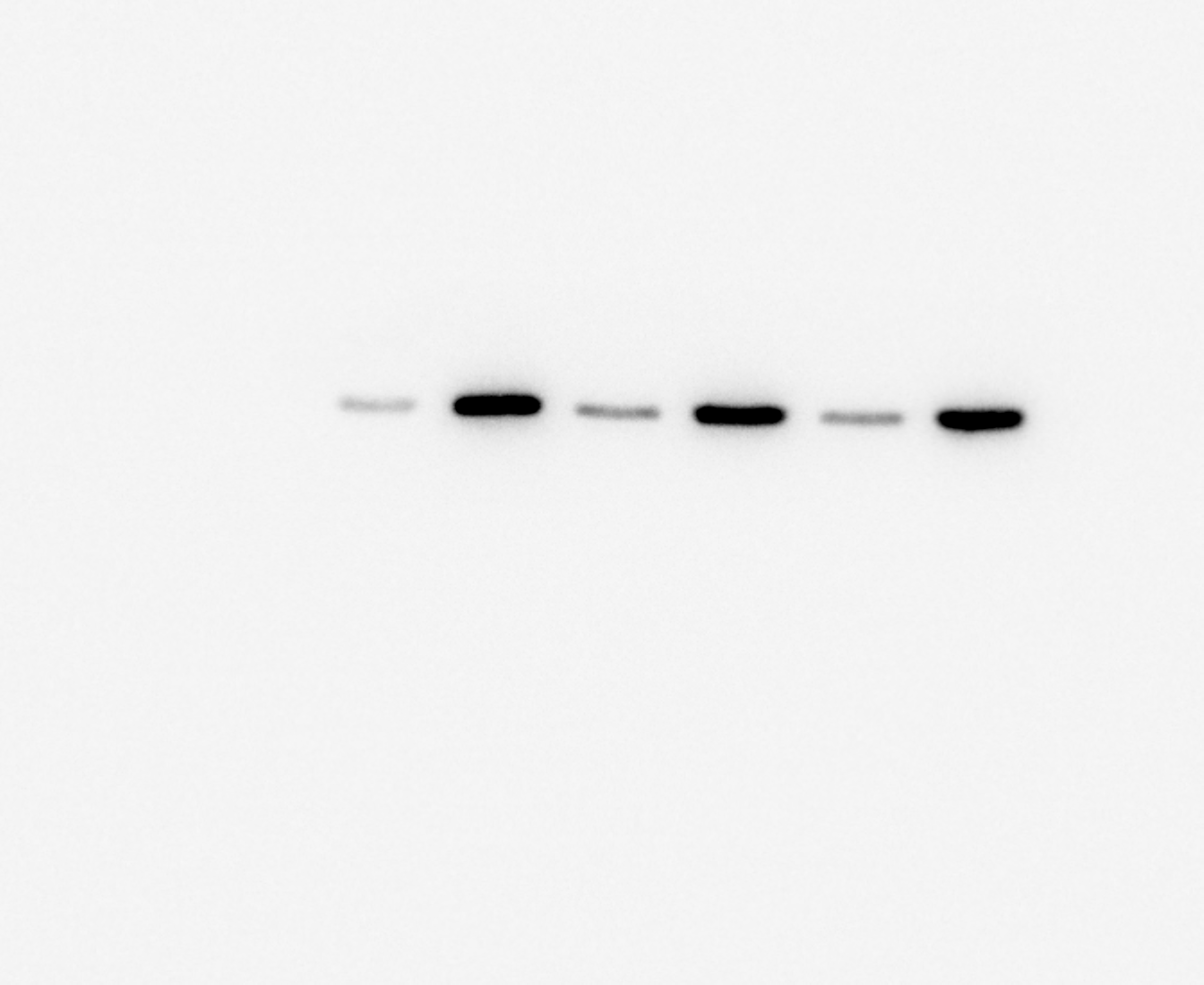

Supplement: Figure 7—source data 2. — The lower membranes correspond to CON group and higher membranes correspond to CLA group. [file elife-99790-fig7-data2.zip › Figure 7-source data 2/FABP4.tif]

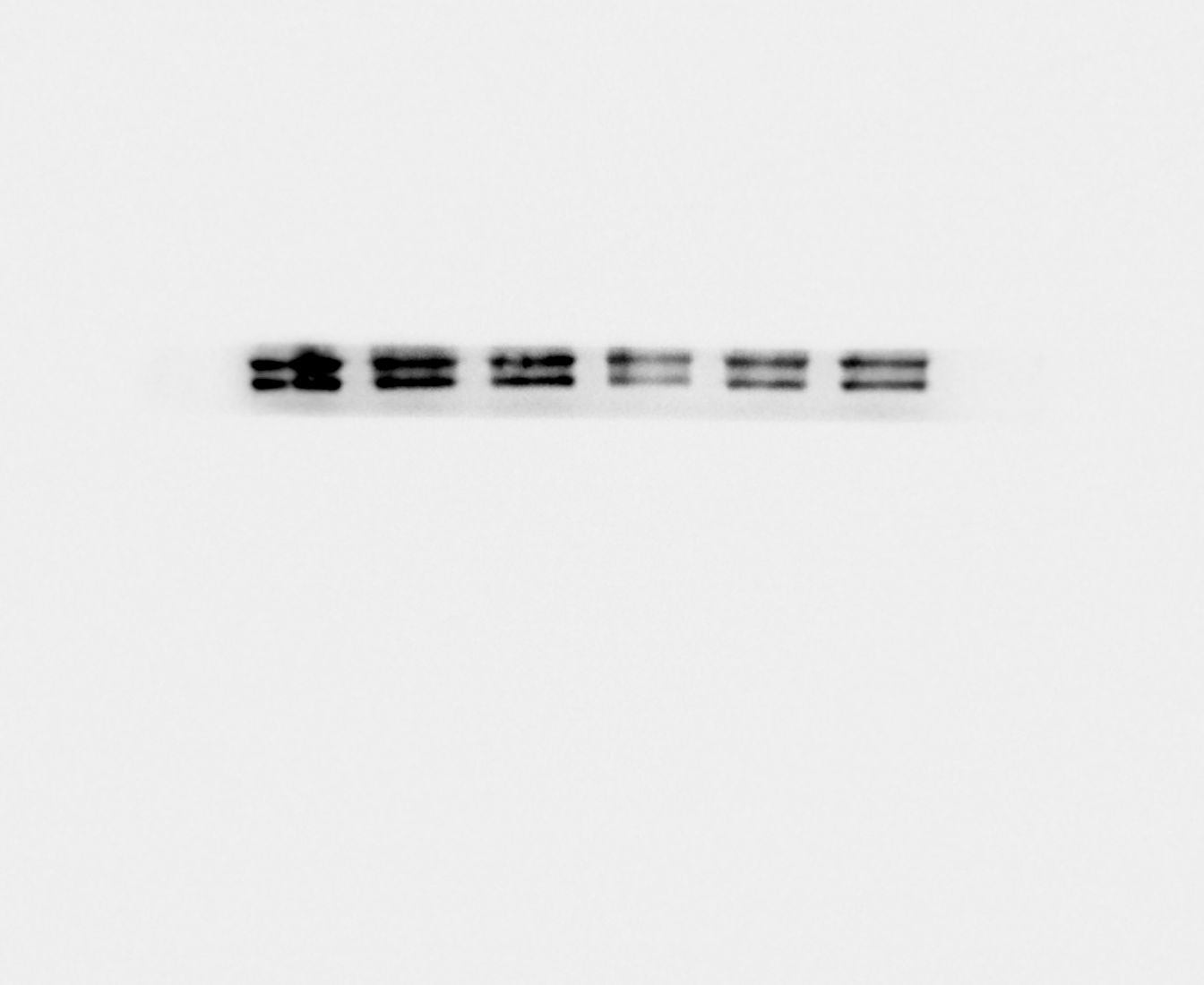

Supplement: Figure 7—source data 2. — The lower membranes correspond to CON group and higher membranes correspond to CLA group. [file elife-99790-fig7-data2.zip › Figure 7-source data 2/JNK123.tif]

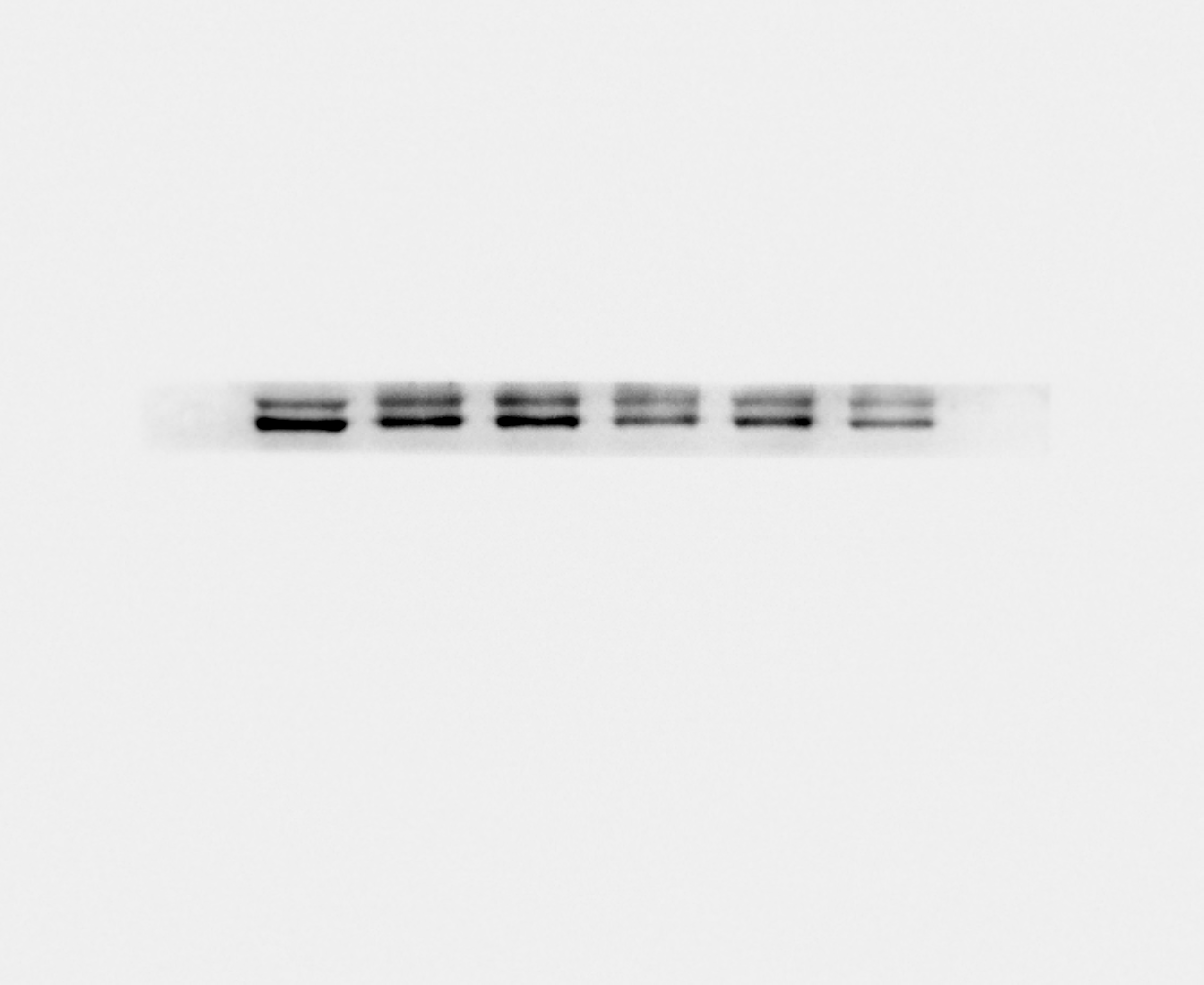

Supplement: Figure 7—source data 2. — The lower membranes correspond to CON group and higher membranes correspond to CLA group. [file elife-99790-fig7-data2.zip › Figure 7-source data 2/P-JNK123.tif]

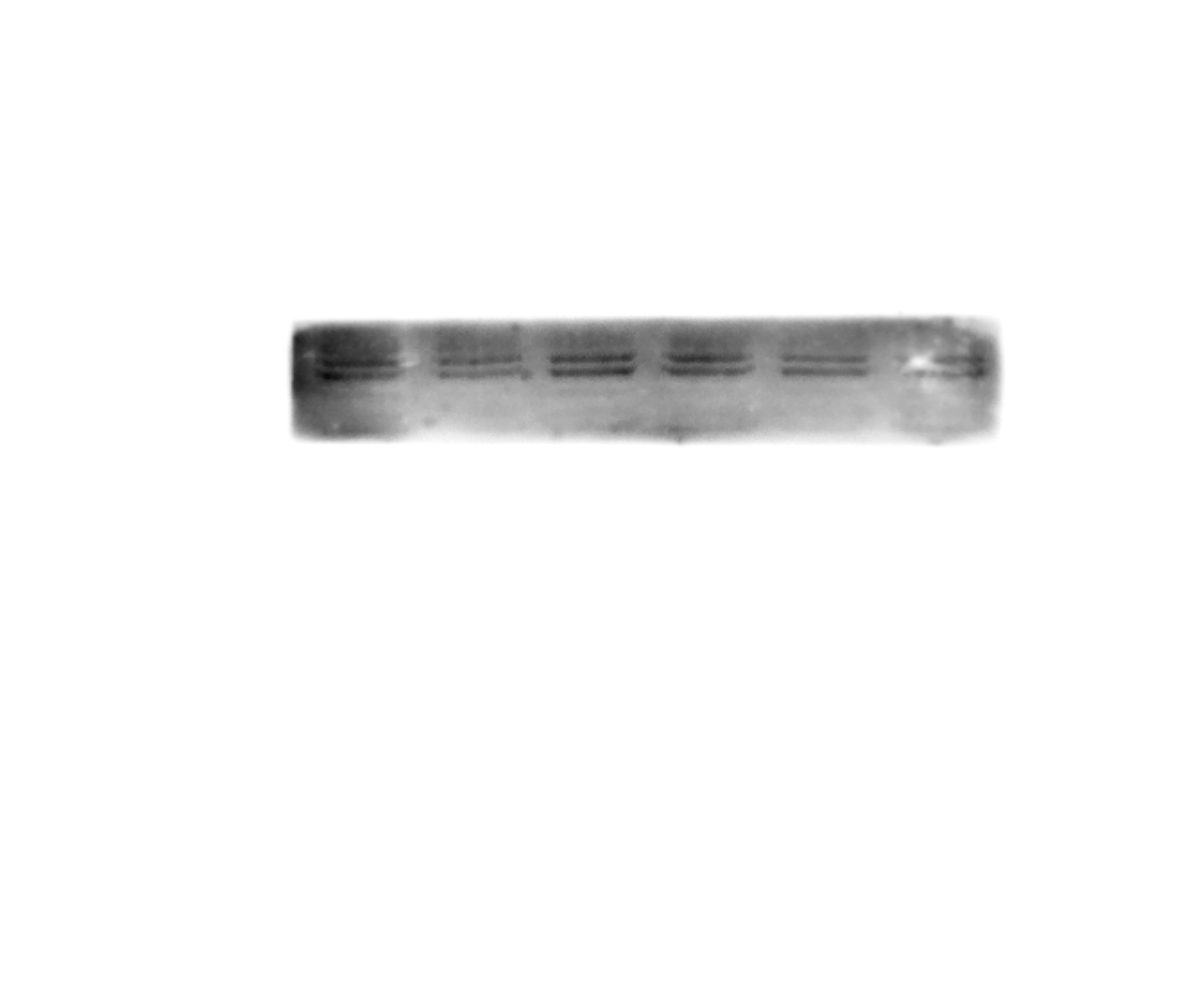

Supplement: Figure 7—source data 2. — The lower membranes correspond to CON group and higher membranes correspond to CLA group. [file elife-99790-fig7-data2.zip › Figure 7-source data 2/PDE4D.tif]

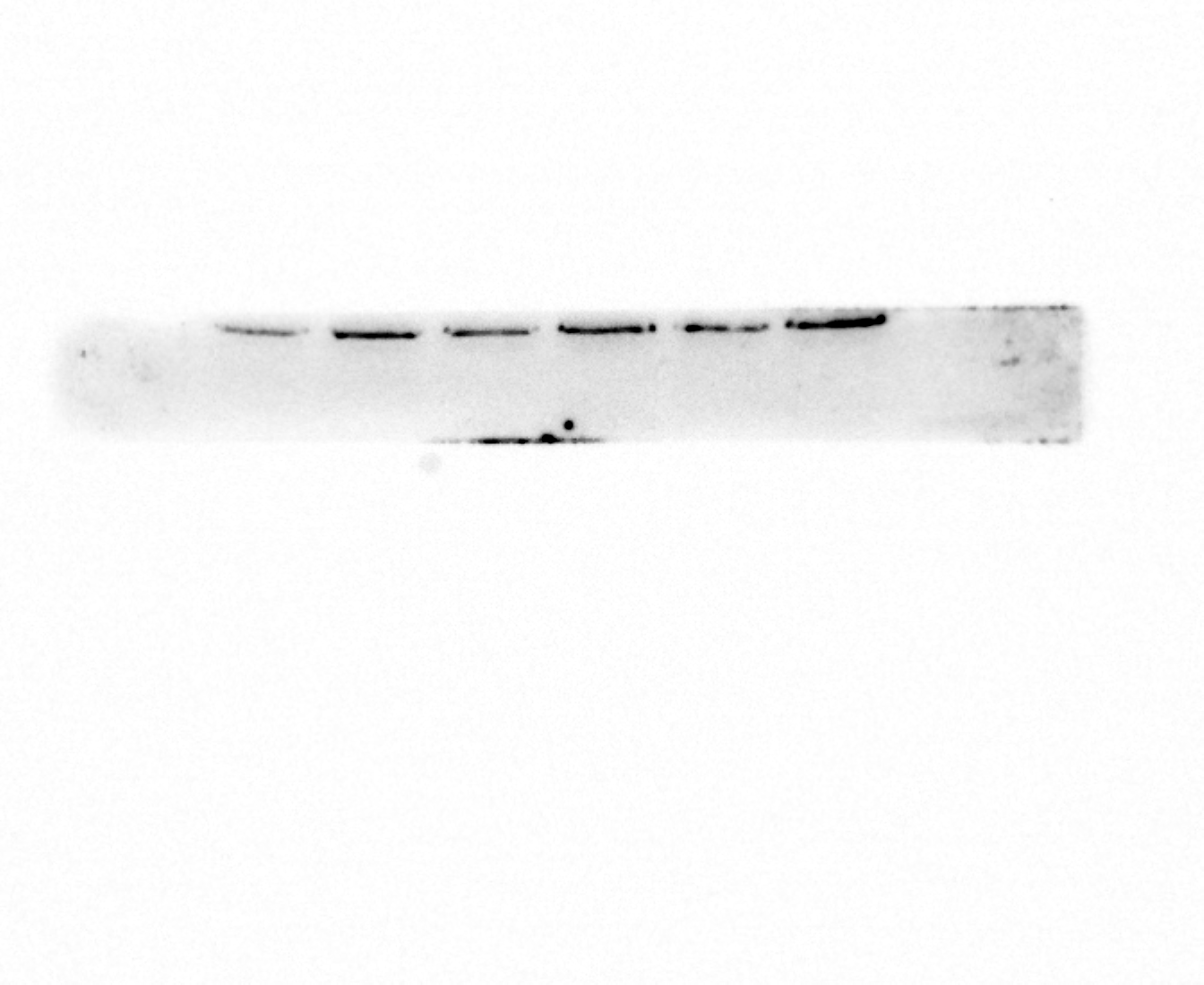

Supplement: Figure 7—source data 2. — The lower membranes correspond to CON group and higher membranes correspond to CLA group. [file elife-99790-fig7-data2.zip › Figure 7-source data 2/SCD1.tif]

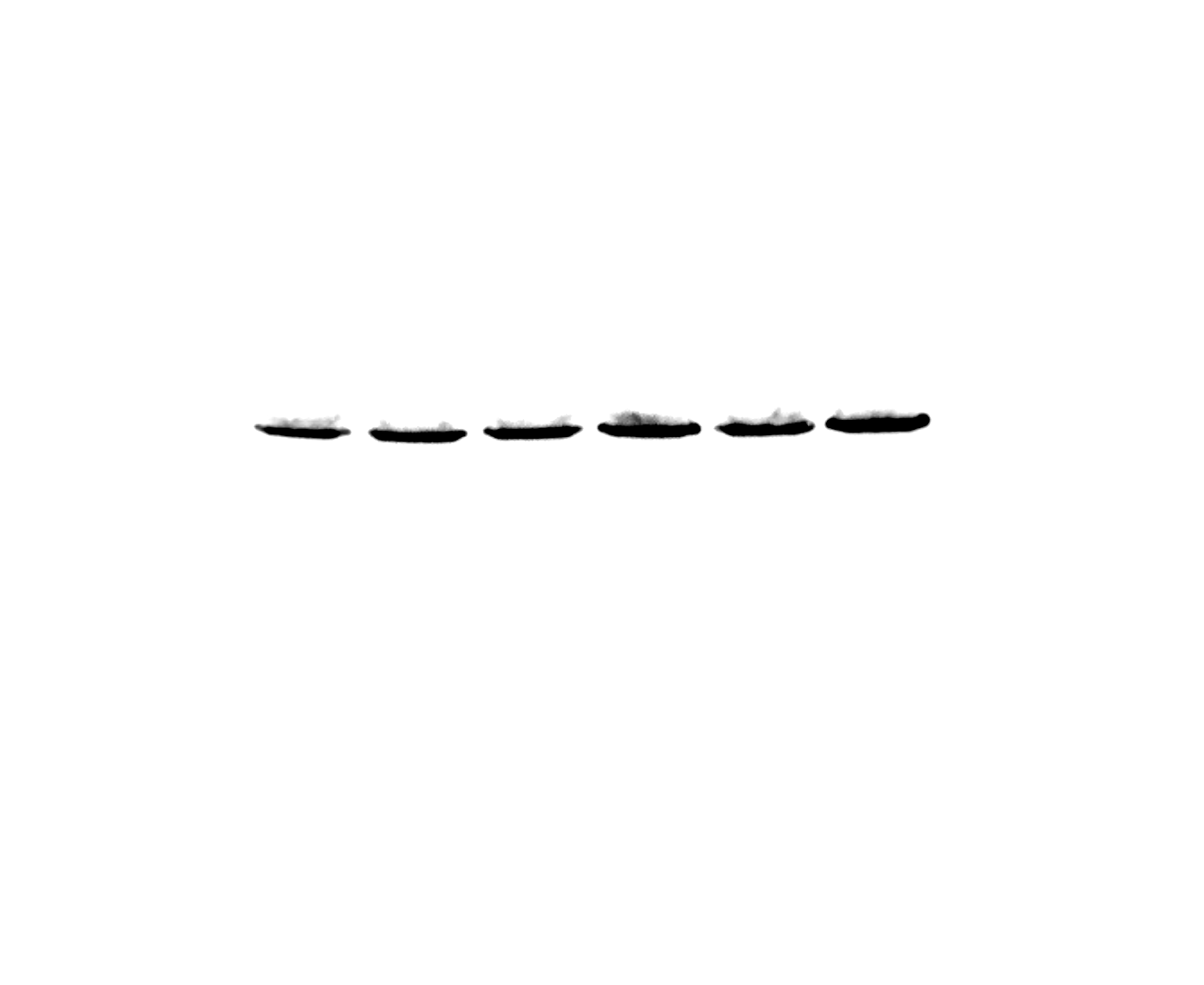

Supplement: Figure 7—source data 2. — The lower membranes correspond to CON group and higher membranes correspond to CLA group. [file elife-99790-fig7-data2.zip › Figure 7-source data 2/β-actin-1.tif]

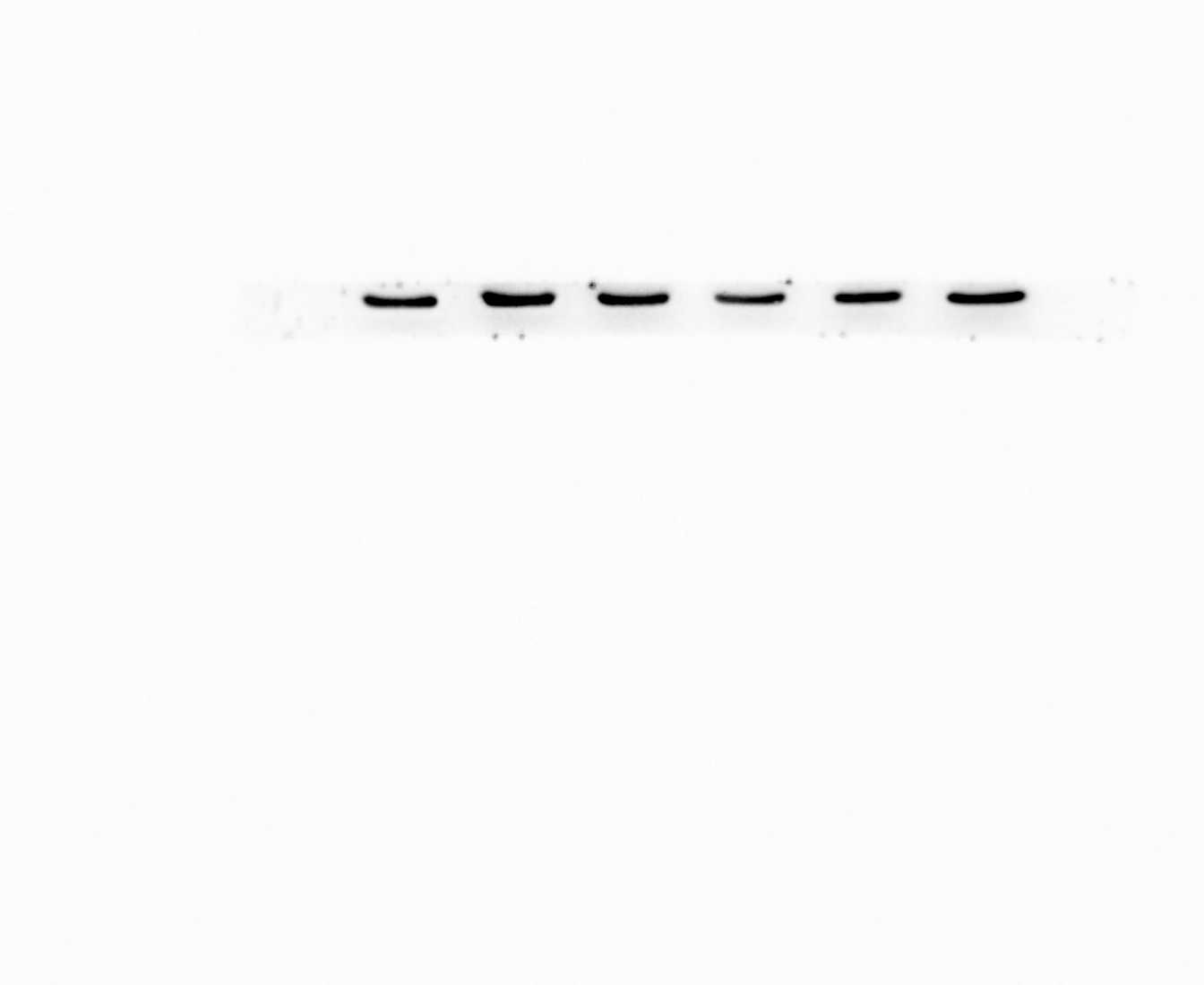

Supplement: Figure 7—source data 2. — The lower membranes correspond to CON group and higher membranes correspond to CLA group. [file elife-99790-fig7-data2.zip › Figure 7-source data 2/β-actin-2.tif]
