# Supplementary material for: Single-nucleus transcriptomics reveal the cytological mechanism of conjugated linoleic acids in regulating intramuscular fat deposition
Source: eLife. 2025 Mar 7;13:RP99790. doi: 10.7554/eLife.99790 (PMC11888599; doi:10.7554/eLife.99790)
Supplement: Supplementary file 1. [file elife-99790-supp1.docx]

**Supplementary File 1.** The primer sequence of qPCR.

| Primer name | Sequence (5'-3') |
| --- | --- |
| 18s-F | CCCACGGAATCGAGAAAGAG |
| 18s-R | TTGACGGAAGGGCACCA |
| ACACA-F | AGCAAGGTCGAGACCGAAAG |
| ACACA-R | TAAGACCACCGGCGGATAGA |
| ADIPOQ-F | CTCCTTCCACGTCACGGTCT |
| ADIPOQ-R | CCAGATAGAGGAGCACAGAGCC |
| ANO4-F | GGTCTGAATCGTCTGCTTACTAATGG |
| ANO4-R | TCCCTTGTGAAGTCCTTTCCTAGAG |
| ARBB1-F | GAACTCCGTGCGTCTGGTCATC |
| ARBB1-R | AGGAACTGCCTGGTGGTCTCG |
| ATGL-F | GCACCTTCATTCCCGTGTAC |
| ATGL-R | TTGTCTGAGATGCCACCGTC |
| COX1-F | AACTGACTCGTACCGCTAATAATCG |
| COX1-R | GGATGCCAGAAGTAATAGGAAGGATG |
| DGAT2-F | AGGACATTGACCTCTACCATGC |
| DGAT2-R | CAGTTCACCTCCAGGACCTC |
| ELOVL6-F | AGAACACGTAGCGACTCCGAAGAT |
| ELOVL6-R | GACATGCCGACCGCCAAAGATAA |
| FABP4-F | TGGAAACTTGTCTCCAGTG |
| FABP4-R | GGTACTTTCTGATCTAATGGTG |
| FABP5-F | ACTGTCTGCGACTTTACCAATGG |
| FABP5-R | TTCTTGTGATTGTGCTCTCCTTCC |
| FASN-F | GCAGGCGCGTGATGGGAATGGTG |
| FASN-R | GCCCGAGCCCGAGTGGATGAGCA |
| HSL-F | CCCCCGTGCGCTGGAGGAGT |
| HSL-R | GGGAGGGGGAGGCGGCAGAC |
| MYBPC1-F | CTATTCTCAGCCCATTCTCGTG |
| MYBPC1-R | TCTGGTCTTGGTTTTCCCTG |
| NEB-F | AGGAAGCAATAGGACAAGGAAC |
| NEB-R | CAATCTCTGGAGTCACAGTGG |
| PDE4D-F | GGAAGATGGCGAGTCAGATACG |
| PDE4D-R | TGGCTCTCCTCCTCCTCTCC |
| PDE7B-F | CCTACATCGTGGAGCCACTCTTC |
| PDE7B-R | TGCTACCGCTGCTGCTTCTG |
| PPARγ-F | GGCGAGGGCGATCTTGACAG |
| PPARγ-R | GATGCGAATGGCCACCTCTTT |
| SCD-F | CAAACACCCAGCCGTCAAAG |
| SCD-R | CGAAGAAAGGTGGCGACGAA |
| SIAH1-F | TTAATCTTCCTGGTGCTGTTGACTG |
| SIAH1-R | GCTTGCTTGCGTGTTCCTATCAG |
| SREBP1-F | GTGCTGGCGGAGGTCTATGT |
| SREBP1-R | AGGAAGAAGCGGGTCAGAAAG |
| THBS1-F | CTGGACTTGCTGTAGGTTATGATGAG |
| THBS1-R | CATAGAAACGGCTGCTGGACTG |
| TIMP3-F | CCTTTGGCACACTGGTCTACAC |
| TIMP3-R | GGTACTGGTACTTGTTGACTTCTAGC |
